# Autologous blood or autologous serum acupoint injection therapy for psoriasis vulgaris: A protocol for a systematic review and meta-analysis

**DOI:** 10.1097/MD.0000000000020555

**Published:** 2020-06-05

**Authors:** Qiuyue Wang, Mao Li, Xingxin Hu, Qian Luo, Pingsheng Hao

**Affiliations:** Hospital of Chengdu University of Traditional Chinese Medicine, Chengdu, China.

**Keywords:** autologous blood, autologous serum, injection, protocol, psoriasis vulgaris, systematic review

## Abstract

**Background:**

Psoriasis vulgaris (PV) is a refractory and relapsing skin disease that affects the physical and mental health of patients and leads to poor quality of life. Current conventional systemic therapy shows a large side effect, which can not be used for a long time, easy to relapse after drug withdrawal, long-term efficacy is poor. At present, traditional Chinese medicine treatment of psoriasis vulgaris effective, can alleviate symptoms, improve the quality of life, stabilize the condition, prolong the remission period. Whereas, there is no related systematic review and meta-analysis. Thus, we intend to conduct a systematic review and meta-analysis to testify autologous blood or autologous serum acupoint injection therapy for Psoriasis Vulgaris.

**Methods:**

Our systematic review will search all randomized controlled trials for autologous blood therapy of PV, electronically and manually, regardless of publication status and language, until March 19, 2020. Databases include PubMed, EMBASE, Web of Science, Cochrane Controlled Trials Register, China National Knowledge Infrastructure, China Biomedical Literature Database, Chinese Science Journal Database, and Wanfang database. Other sources, including reference lists of identified publications and meeting minutes, will also be searched. Manually search for grey literature, including unpublished conference articles.

**Result:**

The main outcomes contain the variation of Psoriasis area and severity index, dermatology life quality index, itching score, the effective rate and adverse events from baseline to the end of studies. This study will provide a comprehensive review of the available evidence for the treatment of PV with this therapy.

**Conclusion:**

We will summarize sufficient evidence to confirm the therapeutic effect and safety of autologous blood or autologous serum acupoint injection therapy for PV. Due to the data is not individualized, formal ethical approval is not required.

**INPLASY registration number:**

INPLASY202040052.

## 1 Introduction

### 1.1 Psoriasis pathogenesis and treatment

Psoriasis is a chronic, immune-mediated, inflammatory, polygenic skin disease with a strong genetic predisposition, and autoimmune pathogenic traits. Affecting approximately 2% of the population in the world. About 90% of psoriasis cases are psoriasis vulgaris (PV). Its typical clinical manifestations are clearly demarcated, erythema, pruritus plaque, covered with silver scales. Plaques can merge and cover large areas of the skin. Common locations include the trunk, the extensor surface of the extremities, and the scalp. PV is not life-threatening, but has been shown to have a significant impact on the physical and mental health of patients, who often feel depressed, anxious, ashamed, and even suicidal. Although psoriasis is one of the most studied skin diseases, its pathogenesis has not been fully elucidated.^[1,2]^

Some studies and systematic reviews have shown that the prevalence rates of cardiovascular and cerebrovascular diseases, diabetes, breast cancer, inflammatory bowel disease, and chronic kidney diseases are associated with psoriasis.^[3–9]^ At the same time, the damage of psoriasis to patients’ mental health and quality of life should be highly valued, even comparable to cancer, myocardial infarction, and depression.^[10,11]^ Mental health comorbidities such as depression, anxiety, suicidal ideation, or suicidal behavior are not uncommon in patients with psoriasis, and sleep difficulties are common.^[12–14]^ Meanwhile, mental illness may in turn cause and promote the progression of psoriasis, suggesting that there may be an overlapping biological mechanism between psoriasis and psychiatric conditions. Proinflammatory cytokines such as interleukin IL-1 and IL-6 were elevated in patients with psoriasis and depression, suggesting that inflammatory processes may be involved in the progression of both diseases.^[15]^

The hallmark of psoriasis is sustained inflammation that leads to uncontrolled keratinocyte proliferation and dysfunctional differentiation. Histological manifestations of psoriasis plaques are echinoderm hyperplasia (epidermal hyperplasia), which is covered with inflammatory infiltration consisting of dermal dendritic cells, macrophages, T cells, and neutrophils. New blood vessels are also a prominent feature.^[16]^ At present, the pathogenesis of psoriasis is still unclear, and the main research focuses on different T cell subsets such as Th1, Th17, and related cytokines such as IL-17, IL-21, IL-22, IL-6, TNF-α, and chemokines. At the same time, the role of IL-23/IL-17 axis in psoriasis is also a hot research topic.^[16]^ In summary, psoriasis is now more recognized as an immune-mediated inflammatory disease.

PV is a chronic recurrent disease that often requires long-term treatment. At present, most of the patients due to the lack of long-term effective treatment, so the disease is serious and prone to relapse, which further increases the medical costs of the patients. A cohort study showed that PV has a significant impact on patients’ income and employment.^[17]^ The choice of treatment for psoriasis depends on the severity of the disease, complications, symptoms, and needs of the patient. Patients with PV are typically divided into 2 categories: mild to moderate to severe plaques, depending on the clinical severity of the lesion, the percentage of affected body surface area, and the patient's quality of life.^[18]^ Clinical disease severity and response to treatment can be graded by many different scores, and the Psoriasis Area and Severity Index (PASI) score has been widely used in PV clinical trials. According to the current US Psoriasis Treatment Guide, there are 5 main therapies for this disease: traditional systemic therapy, biologics, topical therapy, phototherapy, and photochemotherapy.^[19]^ Methotrexate, cyclosporine A and retinoic acid are the traditional systemic treatment options for psoriasis. Although the above drugs are currently used as the most cost-effective first-line drugs, their potential side effects and teratogenic toxicity should not be ignored. Due to the common side effects involving mainly the kidney and the liver, such as nausea, leucopenia, Hypertension, toxicity, and liver transaminase elevation.^[16]^ Topical drugs are currently internationally recognized as topical steroids. Vitamin D analogues and salicylic acid are also recommended in combination with topical steroids. But the side effects of topical steroids are a frequent topic of discussion between doctors and patients. Patients are often most concerned about potential side effects associated with “steroid use”.^[20]^ In the past few years, the accelerated development of the treatment of psoriasis has led to the development of advanced targeted biologic drugs. Currently, drugs targeting TNF-α, IL-23, and IL-17 and signaling pathways such as JAK/STAT are effective in the clinical treatment of PV.^[16]^ However, adverse reactions caused by biological agents have been frequently reported, such as serious infection, hepatitis B and C virus reactivation, interstitial pneumonia, drug-induced lupus erythematosus, etc. More clinical studies are needed on the safety of bio targeted therapy.^[21]^ Moreover, it is expensive and not fully marketed and covered by medical insurance in China, so it is not a good choice for PV patients in China. PV is a complex multifactorial disease. Despite the emergence of many new treatments in recent years, PV is still a treatable but as yet incurable disease. In China, we have been exploring the methods under the guidance of traditional Chinese medicine theory, such as Chinese Herbs, acupuncture, and moxibustion to treat PV patients, with good efficacy, low recurrence rate, and the economy as our pursuit.

### 1.2 Description of the intervention

Autogenous blood (AB) or autogenous serum (AS) acupoint injection therapy is an acupuncture-related technique that specifically separates the whole blood or serum from the patient's own venous blood and then re-infuses it into the patient's own muscle tissue or acupoint such as Zusanli (ST36) for treatment. According to ancient Chinese medical theory, stimulation of acupuncture points is an effective method of treatment, used to prevent diseases and improve health. Now more and more researches are proving that stimulating acupuncture points can regulate the immune system. The effect of emission on diseases is produced by motivating the inherent regulatory system in the body, having the characteristics of whole regulation, dual-directional regulation, etc. The modern scientific researches show that the body's inherent regulatory system is neuro – endocrine – immune network.^[22]^ As mentioned above, psoriasis is an immune-mediated inflammatory disease. Some systematic reviews have proved that acupoint stimulation has a therapeutic effect on psoriasis, and acupuncture can be used as a complementary and alternative medicine therapies for psoriasis.^[23–25]^ Zusanli (ST36) is the most commonly used acupoint for AB or AS acupoint injection therapy in clinical practice. And effective stimulation of Zusanli (ST36) has the effect of immune regulation and inhibition of inflammation.^[26,27]^ AB or AS acupoint injection therapy has been recommended for the treatment of allergies, inflammation, infections, and autoimmune diseases, including acne,^[28]^ chronic urticaria,^[29]^ chronic obstructive pulmonary disease,^[30]^ and diabetes,^[31]^ etc. It can also be used safely in pregnant or lactating patients.^[32]^ The account of it is cheap, easy to use and safe. Therefore, AB or AS acupoint injection therapy may be an option for treating patients with PV in developing countries.

## 2 Objectives

The purpose of this systematic review is to comprehensively collect high-quality randomized controlled trials (RCTs), analyze and summarize the evidence to evaluate the efficacy and safety of AB or AS acupoint injection therapy for PV. To provide evidence for clinical practice by evaluating the efficacy and safety through the Cochrane systematic evaluation. We may recommend an effective treatment that provides clinicians with clinical decision making options to help patients seek further treatment options.

## 3 Methods and analysis

### 3.1 Study registration

The protocol for this systematic review was registered on INPLASY and is available in full on the inplasy.com (https://doi.org/10.37766/inplasy2020.4.0052). The registration number is INPLASY202040052 and the DOI number is 10.37766/INPLASY2020.4.0052. This protocol is developed in accordance with the Preferred Reporting Items for Systematic Reviews and Meta-analyses Protocols (PRISMA-P) statement guidelines.^[33]^ The PRISMA Extension Statement is used to ensure all aspects of methods and findings are reported.

### 3.2 Inclusion criteria of study selection

#### 3.2.1 Type of studies

There will be no restrictions on the length of treatment and duration of follow-up. This systematic review will include high-quality RCTs in English or Chinese that evaluated the therapeutic effect and safety of AB or AS acupoint injection therapy for PV. Without any date of dissemination or restriction of publication type. Our systematic review will search all RCTs, electronically and manually, regardless of publication status and language, until March 19, 2020. To RCTs, it should report adequate randomization methods, eligible diagnosis, eligible outcome measurement, and statistical methods description. Blinding will not be a part of the inclusion criteria because of the particularity of acupuncture manipulation. We will exclude the following types of studies: controlled (non-randomized) clinical trials, case reports, observational study, retrospective studies, animal mechanism studies, self-controlled, random crossover studies.

#### 3.2.2 Types of participants

The patient must be at least 18 years and less than or equal to 65 years of age. Gender is not restricted. The stage or severity of the disease is not limited. Psoriasis must be diagnosed according to at least 1 internationally or nationally authorized diagnostic criterion. The international standard refers to the diagnostic criteria for psoriasis in the “Cecil Textbook of Medicine”. Domestic standard refers to the diagnostic criteria for psoriasis in “Skins and Venereology”, “Clinical Dermatology”, or “Integrated Chinese, and Western Medicine Skin Dermatology”. The groups were well balanced when they Were enrolled.

#### 3.2.3 Types of interventions

The intervention group will use AB or AS acupoint injection therapy, while the control group adopts the placebo, drugs (modern medicine or traditional Chinese medicine (TCM)), other TCM therapies such as acupuncture, cupping and so on, or other active treatments, no treatment, diet and exercise therapy. The intervention group includes either a single AB or AS acupoint injection therapy or a combination of AB or AS acupoint injection therapy and main therapies (traditional systemic therapy, biologics, topical therapy, phototherapy, photochemotherapy, and TCM). It does not include the combination of AB or AS acupoint injection therapy with different types of TCM adjuvant therapy (such as acupuncture and moxibustion, TCM decoction, etc).

#### 3.2.4 Outcomes

***Primary outcomes***

1) Measurements were based on the PASI score. Healing: the reduction rate of PASI score after treatment was >90%. Significant effect: the reduction rate of PASI score after treatment was 60% to 89%. Effective: the decline in PASI scores ranged from 20% to 59%. Invalid: PASI score reduction rate <20%. Reduction rate of PASI score = (pretreatment PASI score – post-treatment PASI score)/pre-treatment PASI score × 100%. Total effective rate = (number of recovered cases + number of effective cases + number of effective cases)/total number of cases 100%.
2) The proportion of participants with serious adverse effects. We will use the definition of severe adverse effects from the International Conference of Harmonization of Technical Requirements for Registration of Pharmaceuticals for Human Use, which includes death, life-threatening events, initial or prolonged hospitalization, and adverse events requiring intervention to prevent permanent impairment or damage.

***Secondary outcomes***

1) Quality of life measured by a specific scale. Available validated scales are the Dermatology Life Quality Index, Skindex, Psoriasis Disability Index, or Psoriasis Symptom Inventory (PSI).
2) Itching score.
3) The proportions of participants with adverse effects.

### 3.3 Data sources and search strategy

The systematic review will search all randomized controlled trials (RCTs) by searching the following database: PubMed, EMBASE, Web of Science, Cochrane Controlled Trials Register, and 4 Chinese databases (involve China National Knowledge Infrastructure, Wanfang, Chinese Science Journal Database, China Biomedical Literature Database) with a language limitation of English and Chinese until March 2020. In addition, the related reference lists of identified publications, meeting minutes, gray literature, and unpublished literature for eligible studies which will be searched by us. The search terms included autologous blood, autohemotherapy, self-blood, acupoint, injection, psoriasis, and psoriasis vulgaris.

The specific search strategy performed in PubMed is presented in Table 1.

**Table 1:**
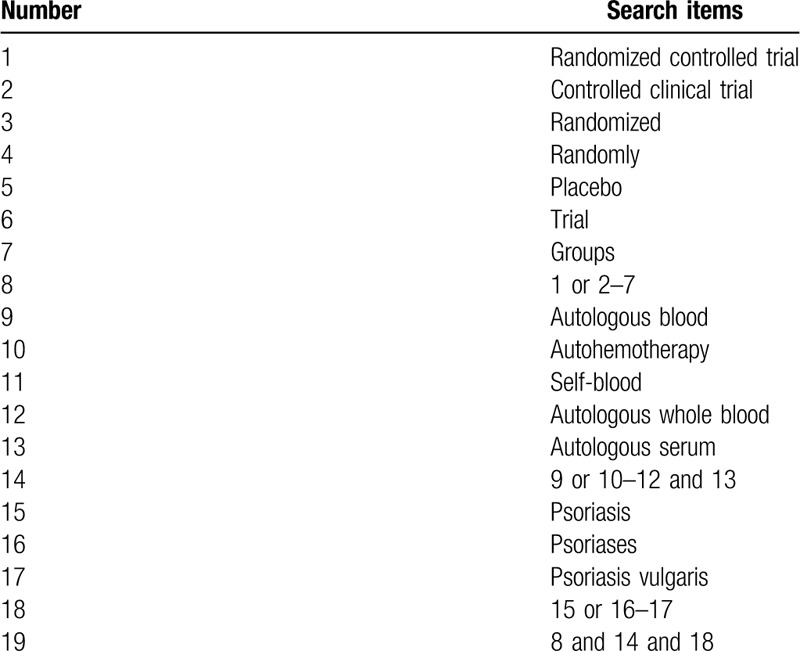
Search strategy in PubMed.

### 3.4 Data collection and analysis

#### 3.4.1 Study selection

**Figure 1:**
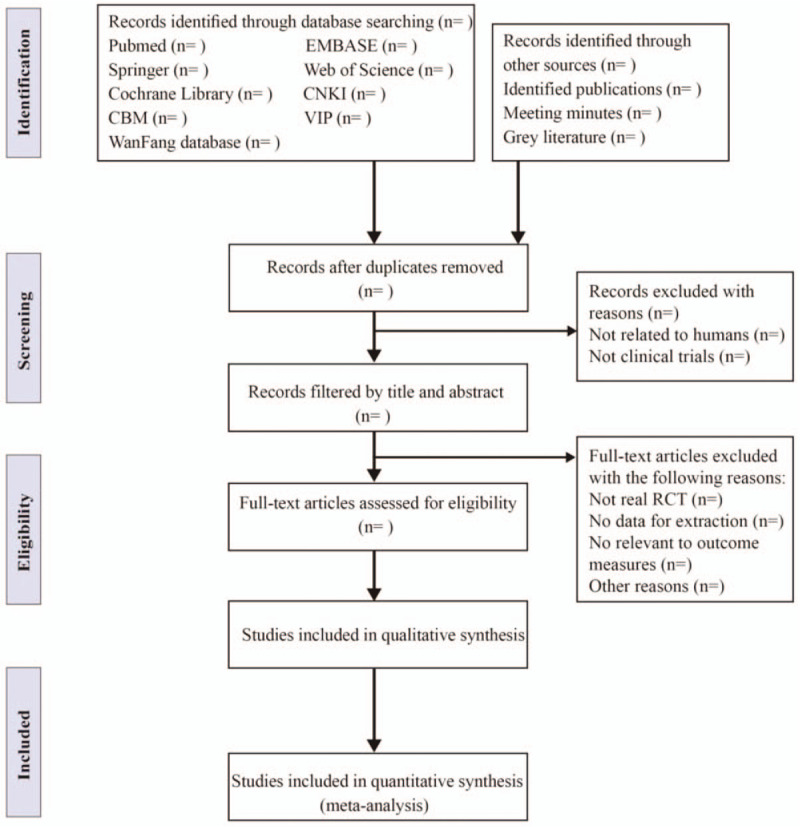
Flow diagram of studies identified.

All potential relevant clinical studies will be screened according to their titles, abstracts, keywords by 2 reviewers (QYW and ML) at the same time independently after removing duplicates and nonclinical trials. And then the intensive reading of full text could authenticate for further assessment if there are studies that could not be clearly included based on both titles and abstracts. Once any disagreement occurs, a decision will be resolved through discussion among the 2 reviewers (QYW and ML), or argument will be adjudicated by a third reviewer (PSH). Details of entire study selection procedure are summarized in flow chart (Fig. 1).

#### 3.4.2 Date extraction and management

Before data extraction, a standard data extraction form containing specified outcomes will be created according to the inclusion. Two reviewers (QYW and ML) will extract data independently from each trial: article general information, participants’ characteristics (such as age, sex, race, and disease history), number of participants on each group, intervention measure of trial and control group, outcomes measures. Any disagreements will be resolved through discussion or consultation between the 2 reviewers if necessary, final determination from a third reviewer (PSH) will be sought. When certain dates are not provided in the paper, we will contact the original author for the needed information.

#### 3.4.3 Risk of bias assessment

Two reviewers (QYW and ML) will evaluate the risk of bias according to the risk of bias (ROB) tool to assess the bias risk of all included studies. We will assess the risk of bias in the following areas: random sequence generation, concealment of allocation sequences, blindness of participants and staff and their result evaluators, incomplete outcome data, selective outcome reports, and other sources of bias. This review will use L, U, and H as the key to these assessments, where L (low) indicates a lower risk of bias, U (unclear) indicates an uncertain risk of bias, and H (high) indicates a higher risk of bias. All reviewers will resolve their differences through discussions. The information contained in the study on the risk of biased assessments will be summarized in a tabular format with a critical discussion of results and impacts. If the information is unclear, we will try to contact the author for the republished articles, and we will only select the original text.

#### 3.4.4 Measures of treatment effect

Data analysis and quantitative data synthesis will be performed using RevMan V.5.3. For continuous data, if there is no heterogeneity, we will use mean difference or standard mean difference to measure the therapeutic effect of 95% CIs. If significant heterogeneity is found, a random-effects model will be used. For the 2-category data, we will use the 95% CIs hazard ratio (RR) for analysis. We will include data from parallel-group design studies for meta-analysis. Only the first phase of the data will be included in the random crossover trial. In these trials, participants were randomly divided into 2 intervention groups and individual measurements for each outcome of each participant were collected and analyzed. The result will be expressed as the RR of the binary data and the standard mean difference of the continuous data.

#### 3.4.5 Unit of analysis issues

Only the first experimental period date will be considered in randomized cross-over trials. In these trials, participants were randomly divided into 2 intervention groups and individual measurements for each outcome of each participant were collected and analyzed.

#### 3.4.6 Dealing with missing data or unclear information

Referring to the Cochrane handbook for systematic reviews of intervention, if there are insufficient details or missing data in relation to the characteristics of the studies, 2 reviewers (QYW and ML) will attempt to contact both senior and/or corresponding author of articles through email or telephone for further information about any missing data or unclear information. If it is not possible to contact the original authors or obtain sufficient information, we will exclude such studies and only analyze the available data and describe it in the discussion. The potential impact of insufficient data on the review results will be took into account in the discussion section.

#### 3.4.7 Assessment of reporting biases

If our review has a sufficient number of included trials that are available in the meta-analysis. If more than 10 trials are included, the funnel plot will be used to assess the reported bias. If the funnel plot is found to be asymmetrical, analyze the cause using the Egger method. We will include all eligible trials regardless of the quality of the method.

#### 3.4.8 Data synthesis and analysis

We will use RevMan V.5.3. for all statistical analyses. If the I^2^ test is less than 50%, a fixed-effect model is used for data synthesis. If the I^2^ test is between 50% and 75%, a random-effects model is used for data synthesis. If the I^2^ test is higher than 75%, we will investigate the possible causes from a clinical and methodological perspective and provide a descriptive analysis or a subgroup analysis.

#### 3.4.9 Subgroup analysis

There is no pre-subgroup plan for this project. When there are significant differences, we will carry out subgroup analysis according to the control group intervention measures and different results.

#### 3.4.10 Sensitivity analysis

When there are enough studies, we will conduct sensitivity analysis of the main results according to the sample size, heterogeneous quality and statistical model (random or fixed effect model) to explore the robustness of the conclusions. Sensitivity analysis will be conducted by removing low-quality studies. If heterogeneity still exists after subgroup analysis, a meta-analysis will be performed again after excluding low-quality tests according to STRICTA checklist. The results of these meta-analyses will be compared and discussed in terms of their sample size, the strength of the evidence, and their impact on the size of the merger effect. However, if there is a high risk of bias in all included studies, we will not conduct sensitivity analysis.

#### 3.4.11 Grading the quality of evidence

We will assess the evidence quality and credibility of the major findings (major outcomes and adverse events) of the studies included in our review, based on recommendations, assessments, development and grading of assessment (GRADE) guidelines.^[34]^ The quality of evidence will be classified as “very low”, “low”, “medium” or “high”. Any differences will be resolved by consensus or with the third review author (PSH).

### 3.5 Ethics and dissemination

The systematic review and meta-analysis do not require to pass the ethics approval, because we include published articles rather than directly adopt interventions in participants. Ultimately, we will publish the results at a peer-reviewed journal follow as our study is completed.

## 4 Discussion

PV is a refractory and relapsing skin disease that affects the physical and mental health of patients and leads to poor quality of life. Current conventional systemic therapy shows a large side effect, can not be used for a long time, easy to relapse after drug withdrawal, long-term efficacy is poor. At present, traditional Chinese medicine treatment of PV effective, can alleviate symptoms, improve the quality of life, stabilize the condition, prolong the remission period.

Domestic and foreign research has proven that alternative treatments such as AB or AS acupoint injection therapy are effective and safe for the treatment of PV, with operability, low-cost, and broad prospects. However, systematic statistical evidence is still lacking. According to the Cochrane method, this study is based on the analysis of clinical RCT evidence at home and abroad, searching and screening the main electronic literature database with evidence-based medical evidence, providing clinicians with more convincing evidence in decision-making, to better guide clinical treatment.

## Author contributions

**Conceptualization:** Qiuyue Wang, Pingsheng Hao.

**Data curation:** Qiuyue Wang, Mao Li.

**Investigation:** Qian Luo.

**Methodology:** Qiuyue Wang, Mao Li.

**Resources:** Qiuyue Wang.

**Software:** Qiuyue Wang, Mao Li.

**Supervision:** Pingsheng Hao.

**Validation:** Xingxin Hu.

**Writing – original draft:** Qiuyue Wang.

**Writing – review & editing:** Pingsheng Hao.
